# A Thick Anastomotic Vein Between Cephalic and Lateral Radial Veins: Clinical Significance of an Unusual Variation

**DOI:** 10.7759/cureus.16629

**Published:** 2021-07-26

**Authors:** Saliha Seda Adanır, Mustafa Orhan

**Affiliations:** 1 Department of Anatomy, Gaziantep University Medicine Faculty, Gaziantep, TUR

**Keywords:** the cadaveric study, cephalic vein, radial vein, venous anastomosis, venous variation, upper extremity

## Abstract

This study presents a thick anastomosis between the cephalic vein and lateral radial vein which, to the best of our knowledge, has not been reported before in the literature. During routine cadaver dissection in the right upper extremity of a 54-year-old male cadaver; in the anterior cubital region, a very thick anastomotic branch was found by piercing the deep fascia, going upwards and laterally, and joining the cephalic vein. Deep dissection results showed that this branch provided an anastomosis between the lateral radial vein and cephalic vein. Despite that variations of superficial veins in the upper extremity are common, the presence of a branch connecting deep and superficial veins is a rare situation. It is well known that veins in the cubital fossa are essential in traditional diagnosis and treatment procedures. Arteriovenous fistula procedure is a widely used method applied by creating a fistula between the cephalic vein or basilic vein and the brachial artery to provide vascular access for hemodialysis in patients with chronic renal failure. Drainage of the lateral radial vein into the cephalic vein through a thick branch, as determined in this case, may disrupt venous drainage in the forearm in an arteriovenous fistula procedure performed between the cephalic vein and the brachial artery. On the other hand, in such a case, if the basilic vein is preferred for this procedure, it is thought that the rate of impairment of venous drainage may be less.

## Introduction

Most of the venous blood of the upper extremity is carried by superficial veins. The cephalic vein (CV) and the basilic vein (BV) are the major superficial veins of the forearm. CV starts from the lateral of the forearm and continues along the upper arm, goes deeper into the deltopectoral groove, and finally joins to the axillary vein. BV, which is in the ulnar part of the dorsal aspect of the forearm, anastomoses with CV through the median cubital vein (MCV). The deep veins of the forearm, namely ulnar veins (UV) and radial veins (RV), are usually found in a pair, accompany the arteries of the same name and join to form the brachial veins (BrV) around the elbow joint [1,2].

The arrangement of the BV and CV in the cubital fossa is highly variable. Anastomoses of BV and CV near the cubital fossa form a pattern similar to an M, Y, or N shape [1-3]. These veins drain into the deep veins at different levels. The BV usually continues as the medial brachial vein and the CV into the axillary vein [4]. Although anastomoses between superficial veins in the cubital fossa are common, superficial and deep vein anastomoses are rare in this region [4-7].

In this study, a cadaveric case with a thick anastomotic branch between the cephalic vein and radial vein is presented. As a result of a detailed literature review, it was found that such a variation had not been reported before.

## Case presentation

**Figure 1:**
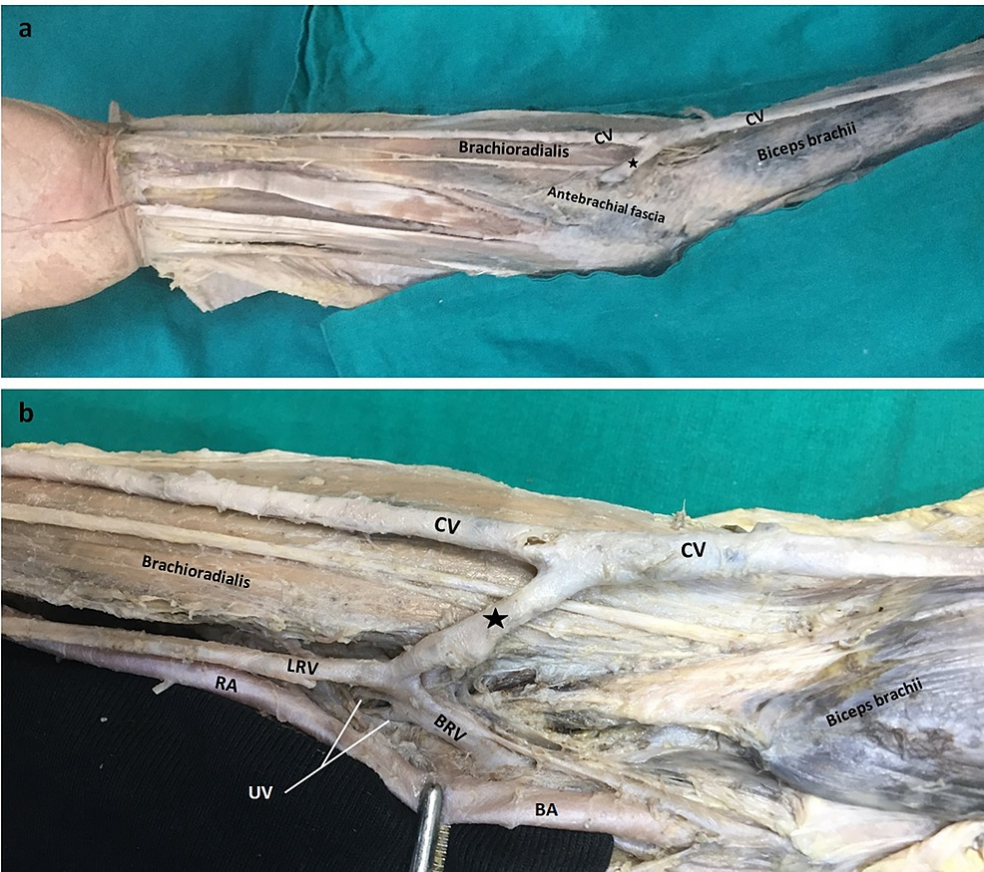
a) Anterior view of the upper extremity, Anastomotic branch (*) that becomes superficial by piercing the deep fascia and joins the CV. b) Anterior cubital region. After the antebrachial fascia is removed, the anastomotic branch (*) deepens and connects with the LRV (lateral radial vein) BA: Brachial artery, BrV: Brachial vein, CV: Cephalic vein, RA: Radial artery, UV: Ulnar vein

During the routine cadaver dissection performed in the laboratory of Gaziantep University Faculty of Medicine, Department of Anatomy, Turkey, in the right upper extremity of a 54-year-old male cadaver, a vessel piercing the deep fascia in the anterior cubital region, traveling 21 mm upward and laterally before joining the CV was observed (Figure 1a). When the deep fascia in the anterior cubital region was removed, it was observed that this vessel separated from the lateral radial vein (LRV) and traveled towards the surface, proceeded medial to the brachioradialis and pierced the deep fascia (Figure 1b).

**Figure 2:**
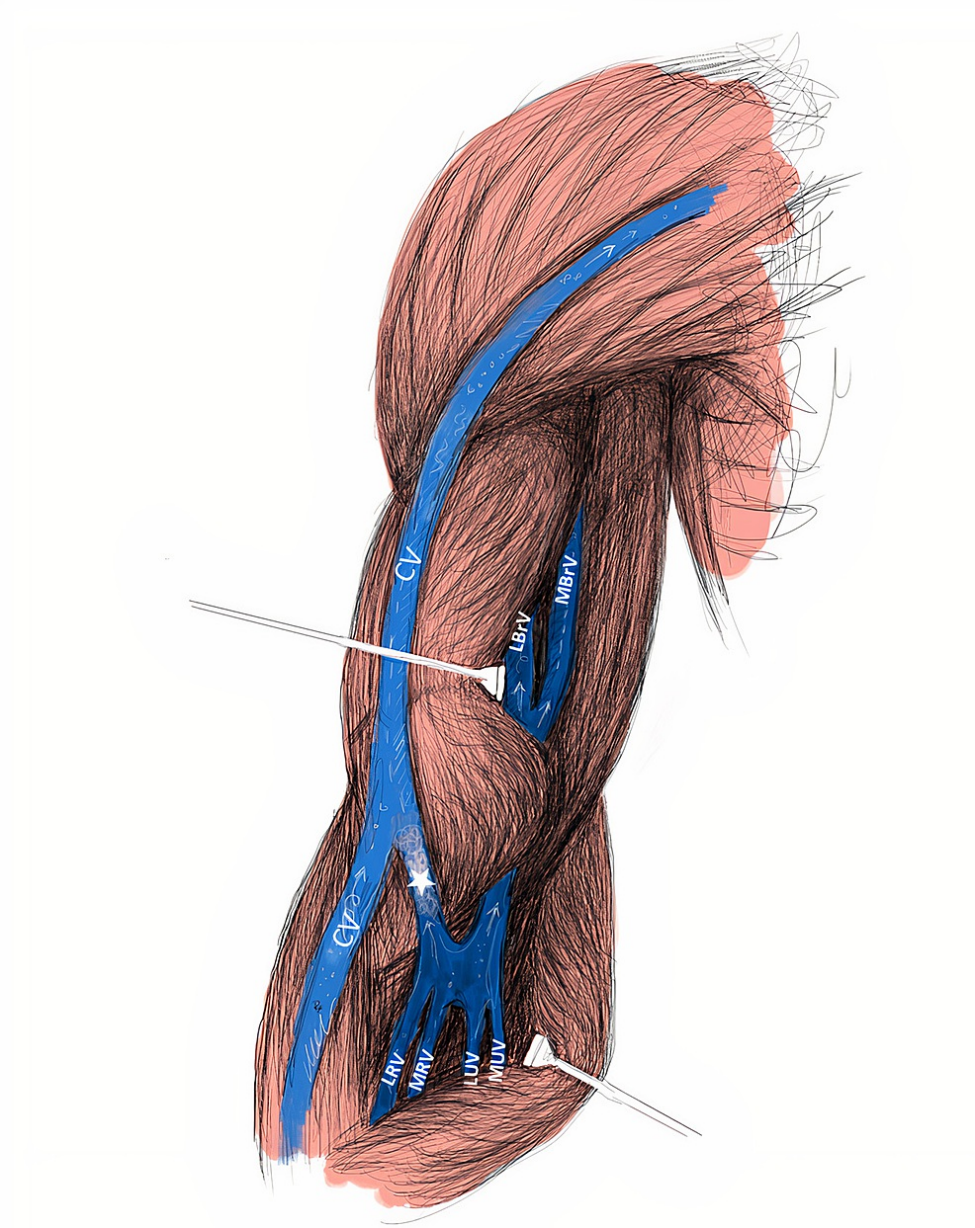
Illustration of the anastomosis (*) between CV (cephalic vein) and LRV (lateral radial vein). MBrV: Medial brachial vein, MRV: Medial radial vein, MUV: Medial ulnar vein, LBrV: Lateral brachial vein, LUV: Lateral ulnar vein

The anastomotic branch originated proximally of the LRV, between the deep vein and the superficial vein, and connected with the CV in the anterior cubital region. Moreover, it was determined that the LRV merged with the medial radial vein (MRV) just above this anastomotic branch and then united with UV and opened to BrV (Figure 2). Regarding vessels diameter in the anterior cubital region, the anastomotic branch's diameter (5 mm) was larger than CV's (4.1 mm), lateral branchial vein's (4.4 mm), and LRV's diameter (2.6 mm). Moreover, MCV was not observed at this extremity, while BV was in its normal course in the forearm and had no connection with CV. In addition, the aforementioned variation was found unilaterally.

## Discussion

Vasculogenesis begins with the formation of blood islets in the vitellus sac and head mesenchyme after gastrulation for the first time. Veins in the body develop from three main pairs of veins, vitelline, umbilical, and cardinal [1,8]. Superficial veins are the first system to grow in the embryo, and initially, deep veins open into superficial veins. Later, with muscle mass development, the deep veins become the main system and the superficial veins drain into the deep veins. In the hand, the fetal pattern continues throughout life and superficial veins are dominant. In the forearm, the pattern in the fetal period changes later. Deep veins become more dominant in the arm and shoulder [4].

Although the variations in the superficial veins of the upper extremity are common, variations in which vessels connecting the superficial veins and deep veins of the upper extremity are seen are rare. These vessels that provide connections between superficial veins and deep veins have been reported as thin perforating branches [9,10]. In a cadaver study by Mikuni et al. [9], whereas it was stated that there were thin perforating branches that supply anastomosis between the superficial veins and deep veins in the forearm, the incidence of these branches was not reported. When another study conducted by Vasudha [10] was examined, it was seen that CV and BV anastomose with deep veins through perforating veins in two out of 50 (4%) extremities. Still, it was not stated that they anastomose with which deep vein. In this study, the presence of a very thick branch that provides anastomosis between superficial and deep veins in the anterior cubital region was shown. In the literature, it can be obviously seen that while perforating branches between superficial and deep veins were mentioned before, the presence of a thick branch as reported in this study was not.

In a meta-analysis study conducted by Yammine and Erić [11] in which 27 different studies were examined, it was reported that the incidence of the pattern that there was no connection between CV and BV was between 4-11%. In addition, it has been reported that this type of pattern is more common in women than men [11]. In this case, similarly, it was found that there was no direct connection between CV and BV and CV provided anastomosis with LRV through a thick vein.

It is known that veins in the cubital fossa are frequently used in clinical procedures such as venipuncture, transfusion, infusion, cardiac catheterization, and vascular access for dialysis, and these veins are significant in traditional diagnosis and treatment procedures [6,12,13]. The arteriovenous fistula (AVF) procedure is a common technique used to gain vascular access in patients with chronic renal failure. In this method, a fistula is created between the CV or BV and the brachial artery [12,14]. Alamshah [14] reported that the rate of deterioration of venous drainage might be lower in an AVF procedure performed in a pattern where the connection between the deep veins in the forearm and MCV is observed. As defined in this case, drainage of LRV one of the deep veins of the arm, into the CV through a thick branch may impair venous drainage in the forearm in an AVF procedure performed between the CV and the brachial artery. On the other hand, in such a case, if BV is preferred for AVF procedure, it is thought that the rate of deterioration of venous drainage may be less.

## Conclusions

In this case report, the presence of a thick branch that provides anastomosis between CV and LRV is reported. As far as we know the existence of such a thick vein has not been reported in the literature so far. It is crucial to master the normal anatomy and variations of superficial veins in the cubital fossa for many clinical procedures. It is thought that the fact that the clinicians are aware of the rare variations can increase the success rate and minimize the complications that may occur in the procedures to be performed in this region.
